# Epac1-deficient mice have bleeding phenotype and thrombocytes with decreased GPIb**β expression**

**DOI:** 10.1038/s41598-017-08975-y

**Published:** 2017-08-18

**Authors:** Gyrid Nygaard, Lars Herfindal, Kathrine S. Asrud, Ronja Bjørnstad, Reidun K. Kopperud, Eystein Oveland, Frode S. Berven, Lene Myhren, Erling A. Hoivik, Turid Helen Felli Lunde, Marit Bakke, Stein O. Døskeland, Frode Selheim

**Affiliations:** 1 Department of Biomedicine, University of Bergen, Bergen, Norway; 2 The Proteomics Unit at the University of Bergen, Bergen, Norway; 3 Centre for Pharmacy, Department of Clinical Science, University of Bergen, Bergen, Norway; 4 Hospital Pharmacies Enterprise, Western Norway, Bergen, Norway; 5 Department of Clinical Science, University of Bergen, Bergen, Norway; 6 Department of Immunology and Transfusion Medicine, Haukeland University Hospital, Bergen, Norway

## Abstract

Epac1 (Exchange protein directly activated by cAMP 1) limits fluid loss from the circulation by tightening the endothelial barrier. We show here that Epac1^−/−^ mice, but not Epac2^−/−^ mice, have prolonged bleeding time, suggesting that Epac1 may limit fluid loss also by restraining bleeding. The Epac1^−/−^ mice had deficient *in vitro* secondary hemostasis. Quantitative comprehensive proteomics analysis revealed that Epac1^−/−^ mouse platelets (thrombocytes) had unbalanced expression of key components of the glycoprotein Ib-IX-V (GPIb-IX-V) complex, with decrease of GP1bβ and no change of GP1bα. This complex is critical for platelet adhesion under arterial shear conditions. Furthermore, Epac1^−/−^ mice have reduced levels of plasma coagulation factors and fibrinogen, increased size of circulating platelets, increased megakaryocytes (the GP1bβ level was decreased also in Epac1^−/−^ bone marrow) and higher abundance of reticulated platelets. Viscoelastic measurement of clotting function revealed Epac1^−/−^ mice with a dysfunction in the clotting process, which corresponds to reduced plasma levels of coagulation factors like factor XIII and fibrinogen. We propose that the observed platelet phenotype is due to deficient Epac1 activity during megakaryopoiesis and thrombopoiesis, and that the defects in blood clotting for Epac1^−/−^ is connected to secondary hemostasis.

## Introduction

Damage to blood vessel walls initiates a series of responses to limit blood loss. At the sites of vascular injury extracellular matrix (ECM) proteins become exposed to blood and react with specific platelet receptors to induce a multistep platelet adhesion process^1–4^. At the shear rates in small arteries and microvasculature, the adhesion depends crucially on the GPIb-IX-V platelet receptor´s interaction with ECM-bound von Willebrand factor (vWF)^2^. This interaction has a rapid on-off-rate causing platelets to roll along the damaged vessel wall, effectively slowing down the platelet flow rate. This facilitates firm adhesion by allowing time for the GPVI, α2β1, α5β1 and α6β1 receptors to bind effectively to their ligands^1^. The integrin receptor αIIbβ3 mediates platelet-platelet interactions by binding to vWF, fibrinogen, fibrin and/or fibronectin^3^. Hence, platelets aggregate and form a primary hemostatic plug to prevent blood loss into the extravascular space. Conditions with qualitative or quantitative defects in vWF, GPIb-IX-V or αIIbβ3 may cause the bleeding disorders von Willebrand’s disease, Bernard-Soulier syndrome (BBS), and Glanzmann’s thrombasthenia, respectively^3, 4^.

The exchange proteins directly activated by cAMP (Epacs) belong to the family of specific guanine nucleotide exchange factors (GEFs) for Ras-like small GTPases. The two Epac isoforms, Epac1 and Epac2, act as sensors for the intracellular levels of the second messenger cAMP. Upon binding of cAMP they become capable of changing downstream targets Rap1 and Rap2 from the inactive GDP to the active GTP-form. The Epac proteins are involved in the regulation of several processes, such as cellular differentiation, secretion, adhesion, and proliferation, and our group has recently shown that Epac1 limits fluid loss from the circulation by tightening the endothelial barrier^5, 6^. Before the discovery of Epac in 1998, many of the processes controlled by Epac were attributed to the cAMP-dependent protein kinase A (PKA)^7^. Interestingly, crosstalk between Epac and PKA signaling has been reported; in some instances they operate in concert while in others they exert opposite effects^8^. To add further complication, Epac1 and PKA have similar affinity for cAMP *in vitro* and can thus respond to similar concentrations of intracellular cAMP^9^.

PKA-mediated cAMP signaling in platelets has been extensively studied and has an inhibitory effect on platelet activation (reviewed in^10^). Although a single article reported trace amounts of Epac1 mRNA and protein in platelets^11^, other and more comprehensive studies, including proteomic and transcriptomic analysis, have not detected platelet Epac1^12, 13^. Furthermore, our group recently demonstrated that the extensively used Epac activator 8-pCPT-2′-O-Me-cAMP completely failed to activate platelet RAP1^14, 15^. Thus, Epac1 is not directly involved in platelet RAP1 activation nor is it likely to be expressed in platelets. However, it has been shown that Epac play a crucial role in hematopoietic cell generation^16^, but its role in thrombosis and hemostasis remains unknown. In the present investigation we have therefore used an global Epac1^−/−^ knockout mouse model for studies on platelet function, *in vivo* assays and quantitative label-free proteomics analyses to determine the potential roles of Epac1 in megakaryopoiesis, platelet activation, and hemostasis. We report here that Epac1^−/−^ mice have increased bleeding time, impaired secondary hemostasis, moderately increased platelet size, increased number of reticulated platelets and significantly changed expressions of several proteins, including up-regulated αIIbβ3-associated ILK and down-regulated plasma coagulation factors. Importantly, Epac1 deficiency also led to generation of megakaryocytes (MK) and platelets with severely reduced levels of platelet GP1bβ, a subunit of the GPIb-IX-V receptor. This may affect the interaction of platelet GPIb-IX-V with the vessel wall at sites of vascular injury, and hence may be translated into defective platelet adhesion and impaired hemostasis under high shear conditions. Our viscoelastic measurements revealed that Epac1^−/−^ mice have a defective clotting process, a result corresponding well with our proteomics data showing reduced plasma levels of factor XIII and fibrinogen. We propose that the observed altered platelet phenotype is due to lack of Epac1 activity during megakaryocyte maturation. The defects in blood clotting for Epac1^−/−^ mice seems to be connected to secondary hemostasis.

## Materials and Methods

### Mouse strains

Mouse models: The Epac-deficient mouse models used in this study are described elsewhere^17^. In short, *loxP* sites were inserted by homologous recombination into the genes encoding Epac1 *(RapGEF3*) and Epac2 *(RapGEF4)* flanking exons 7–10 in *RapGEF3* and exons 12–13 in *RapGEF4*. These exons encode the cAMP-binding domain in both proteins. A Neomycin cassette flanked by *frt*-sites were used for screening purposes and thereafter excised. Epac1^floxed/floxed^ and Epac2^floxed/floxed^ mice were generated at the Mouse Clinical Institute, Strasbourg, France. In this study, mice globally deleted for Epac1 (Epac1^−/−^) or all isoforms of Epac2 (Epac2^−/−^) were produced by crossing floxed animals with mice expressing Cre recombinase from the cytomegalovirus (CMV)-promoter. The mice were bred against a C57BL/6JBomTac (Taconic, Denmark) genetic background for at least ten generations by the start of the study. Epac1^−/−^ and Epac2^−/−^ mice are healthy and apparently indistinguishable from WT mice under standard housing. The wild type C57BL/6JBomTac animals included were littermates and commercial mice (Taconic, Denmark). The animal experiments were approved by the Norwegian Animal Research Authority and conducted according to the European Convention for the Protection of Vertebrates Used for Scientific Purposes. Artificial lighting was maintained on a 12:12 hour light-dark cycle and room temperature was kept constant at 23 °C. All mice were housed in a pathogen-free facility with access to water and chow ad libitum.

### Chemicals and reagents

Isoflurane was from Schering-Plough Animal Health (Kenilworth, NJ). PE Rat Anti-Mouse CD41, FITC RAT Anti-Mouse CD62P and FITC RAT Anti-Mouse CD45 was from BD Biosciences (Franklin Lakes, NJ). Thrombin was from Parke Davis (Morris Plains, NJ) and Type I Collagen (Vitrogen 100) from Angiotech BioMaterials (Palo Alto, CA). All other chemicals and reagents were purchased from Sigma-Aldrich (St. Louis, MO).

### Bleeding time, hemoglobin measurement and whole blood clotting time tests

Mice were kept under isoflurane/O_2_/NO_2_ anesthesia and placed on a heating pad at 37 °C. For bleeding time determination, exactly 5 mm of the distal tip of the tail was amputated using a scalpel and the tail immediately submerged into 10 ml phosphate buffered saline (PBS) at 37 °C. The bleeding end point was the time when bleeding had ceased for at least 10 seconds. Bleeding was also quantified by measuring the resulting hemoglobin content in the PBS solution. For this, 1 ml RIPA buffer was added followed by sonication. Absorbance was read at 575 nm in an ASYS UMV340 plate reader with Digiread 1.2.0.2 software (Biochrom, Cambridge, UK). A whole blood clotting time test was performed on freshly drawn blood as described^18^.

### Preparation of mouse platelets and plasma samples

Blood was obtained from mice euthanized with CO_2_. Between 500–1000 μl was drawn from the left ventricle into a 2 ml syringe, containing 100 μl acid citrate dextrose (ACD), 200 μl Ca^2+^-free Tyrode’s solution (136 mM NaCl, 2.7 mM KCl, 0.77 mM NaH_2_PO_4_ × 2H_2_O, and 2.0 mM MgCl_2_ × 6H_2_O, pH 7.3) 5 mM glucose and 0.05% bovine serum albumin. The blood was centrifuged at 300 × *g* for 5 minutes at room temperature (RT), and the resulting platelet-rich plasma centrifuged at 1000 × *g* for another 10 minutes in the presence of 10 μl ACD. The supernatant was transferred to Eppendorf tubes and the pelleted platelets resuspended in Tyrode’s buffer and adjusted to 3.5 × 10^8^ platelets/ml. For proteomic analyses, platelets were further purified by size-exclusion through Sepharose CL-2B gel (Pharmacia Biotec, Sweden) as described previously^19^.

### Assessment of P-selectin translocation

Isolated mouse platelets (5 μl) were added to polystyrene tubes containing FITC-conjugated anti-mouse CD62P (P-selectin) and PE-conjugated Anti-Mouse CD41 (integrin subunit: αIIb). Various concentrations of agonists (0.035–0.1 U/ml thrombin, 10–40 μM ADP and 20–60 μg/ml collagen) were added and the platelets were incubated for 15 minutes and then fixed in 0.2% paraformaldehyde (PFA). The level of P-selectin translocation was assessed by flow cytometry using a FACSCalibur (BD Biosciences, Franklin Lakes, NJ) and FlowJo software (Tree Star, Inc., Ashland, OR). In brief, 5000 events were conservatively identified as platelets by adjusting forward- and side-scatter thresholds to exclude platelet-derived microparticles (PMPs, identification of PMPs was done as previously described^15^). The thrombopoietic marker CD41 and forward- and side-scatter distributions were then used to confirm the platelet population, before the level of P-selectin surface expression was scored as percentage of CD62P positive platelets.

### Determination of platelet number

Blood (10 μl) from the facial vein of live mice was drawn into ACD coated syringes and then fixed in 0.2% PFA. The fixed whole blood was incubated over night (O/N) with CD41 antibody before platelets were identified by both CD41 positivity and forward- and side- scatter by flow cytometry (FACSCalibur). The platelet populations were gated and data collected for 1 minute. The flow rate of the FACSCalibur combined with the dilution factor of the whole blood in fixative was used to calculate the number of platelets/ml.

### Determination of reticulated platelets

The proportion of reticulated platelets in platelet-rich plasma (PRP) was measured using thiazole orange according to the method described by Hartley *et al*.^20^. A FASC Accuri C6 (BD Biosciences, San Jose, CA, USA, Accuri Cytometry) was used to detect fluorescent platelets, and the data were analysed using the Accuri C6 Software (BD Biosciences). In brief, the proportion if reticulated platelets were determined by adjusting the gating for platelets which were positive for thiazole orange, but negative for the same platelets treated with RNAse A prior to staining. The positive fraction in the RNAse treated samples was subtracted to eliminate false positives.

### Scanning electron microscopy

Platelets were treated with vehicle or 0.07 U/ml thrombin for 10 minutes before fixation and sample preparation as described^21^. Specimens were examined using a Jeol JSM-7400F scanning electron microscope with Jeol PC-SEM 7400 software (Jeol Ltd., Tokyo, Japan).

### Preparation of mouse embryonic liver samples

Mice (8–10 weeks old) were euthanized by CO_2_ at 2 weeks of gestation. The visceral yolk sac containing the embryos was excised from the uterus and immediately submerged in ice-cold PBS. Single embryos were isolated and placed in PBS in petri dishes. The tails were collected for sex determination by PCR using the following primers: Fragile X, sense 5′- GTGGCTTCTTCAACAATCTAACCCTAAT -3′ and antisense 5′- CCATCTCCTGTGCCCTTTTAGCTAT′; UT-A1, SRY, sense 5′- GCCCTACAGCCACATGATATCTTAAAC -3′ and antisense 5′- GAGGCAACTGCAGGCTGTAAAATG -3′. The embryonic livers were collected and fixated in Karnovsky’s solution for 24 h followed by dehydration with graded alcohol solutions. The livers were embedded in Agar100 resin and 1 μm thin sections were cut and stained with toluidine blue.

### Determination of megakaryocyte number and morphology

Mice were euthanized with CO_2_ and bone marrow cells isolated from femurs and tibias, by gently flushing the femoral or tibia interior with PBS into a sample vial^22^. Using flow cytometry (FACS Aria SORP, BD Biosciences, Franklin Lakes, NJ), megakaryocytes were identified by Hoechst33342 (nucleic stain) and positive staining for CD41, as well as negative signal for the erythrocyte marker CD45. For morphological studies, the bone marrow was smeared onto glass slides and stained with May-Grünwald-Giemsa. For quantification of megakaryocytes in mouse liver, the total megakaryocyte count from four to six sections from the same specimen was determined using light microscopy. The megakaryocytes were identified by their large and lobulated nuclei, their size and the pale cytoplasm surrounded by a demarcation membrane^23^. The area of the sections was determined using ImageJ software^24^, and average number of megakaryocytes per mm^2^ was calculated.

### Multiplate and ROTEM whole blood analysis

Murine whole blood samples for rotational thromboelastometry (ROTEM® *delta*, Tem International, Basel, Switzerland) was collected from the left ventricle into a 2 ml syringe as described above for platelet preparation, to a final concentration of 3.2% liquid sodium citrate (VACUETTE, Greiner Bio-One International GmbH, Kremsmunster, Austria). After 7 minutes incubation at 37 °C 300 µL of citrated whole blood was analyzed after manufacturer’s instructions with STARTEM, INTEM, EXTEM and FIBTEM liquid reagents (Tem International, Basel, Switzerland). The measuring time was increased to 90 minutes to obtain higher MCF values, but no other parameters were changed.

For whole blood aggregation analysis on the Multiplate® analyzer (Roche Diagnostics Ltd., Rotkreuz, Switzerland) 2 ml Lithium Heparin blood collection tubes (VACUETTE, Greiner Bio-One International GmbH, Kremsmunster, Austria) were used. After a minimum of 30 minutes resting period in room temperature, 300 µL of whole blood was analyzed after manufacturer’s instructions with ADPtest (Roche Diagnostics Ltd., Rotkreuz, Switzerland).

### Sample preparation for proteomics

Plasma samples (150 μg) were depleted for albumin using the Qproteome Murine Albumin Depletion Kit with spin columns as described by the manufacturer (Qiagen, Germantown, MD). Depleted samples (average 61 μg) were desalted using four volumes of ice-cold acetone and incubated O/N at −20 °C. Protein concentrations in platelet and plasma samples were measured using the BCA Protein Assay Kit (Product # 23225, Pierce, Thermo Scientific, Rockford, IL) using a SpectraMax 96 well plate reader, SoftMax Pro Protein Quantitation and BCA software (Sunnyvale, CA). Platelet proteins (10 μg total in SDS-sample buffer) were separated on a 10% SDS-polyacrylamide gel. The gel was sliced crosswise into 23 bands and digested in-gel with trypsin^25^. The sample preparation is described in detail in the supplementary methods section (S5).

### Mass spectrometry analysis of tryptic peptides using LC-MS

Tryptic peptides were injected into an Ultimate 3000 RSLC system (Thermo Scientific, Sunnyvale, CA) connected online with positive electrospray ionization on a LTQ-Orbitrap Velos Pro mass spectrometer (Thermo Scientific, Bremen, Germany). The tryptic plasma peptides (2.5 μg) were eluted with a 180 min biphasic acetonitrile (I) gradient from a 50 cm analytical column (Dionex #164570, Acclaim PepMap100 nanoViper column, 75 μm i.d. × 50 cm, packed with 3 μm C18 beads (Thermo Scientific)), whereas the tryptic platelet-derived peptides were separated with a 90 min gradient on a 15 cm analytical column (Acclaim PepMap 100, 15 cm × 75 µm i.d. nanoViper column, packed with 2 µm C18 beads). The eluting peptides were ionized in the electrospray and analysed by the LTQ-Orbitrap Velos Pro. The mass spectrometer was operated in the DDA-mode (data-dependent-acquisition) to automatically switch between full scan MS and MS/MS acquisition. The LC-MS analysis is described in detail in the Supplementary methods section.

### Label-free protein quantification and data analysis

The software Progenesis LC-MS® Ver 2.6 (Nonlinear Dynamics Ltd, Newcastle, UK) and SearchGUI/PeptideShaker^26^ were used for plasma protein quantification and identification, respectively. MaxQuant module with Andromeda^27, 28^ (version 1.3.05) was used for platelet protein quantification and identification.

### Data Availability

The raw LC-MS data have been deposited to the Proteome Xchange Consortium via the PRIDE^29^ repository with dataset identifier PXD000282 and null. A detailed description of the label-free data analysis is given in the Supplementary methods section.

### ELISA

The concentration of plasma Von Willebrand factor (vWF) was determined after mice had received an intra-peritoneal (i.p.) injection of 0.1 ml of either vehicle (0.9% NaCl) or the arginine vasopressin (AVP) analog dDAVP (1ng/g bodyweight in 0.9% NaCl). After 1.5 h the mice were euthanized by CO_2_ followed by immediate cardiac puncture to collect 0.4 ml blood into a 0.5 ml syringe with 0.1 ml ACD. Plasma was isolated from blood by centrifugation for 10 minutes at 1000 x *g*, and samples were analyzed using the Mouse Von Willebrand Factor ELISA Kit (antibodies-online Inc., ABIN2540487) according to the manufacturers instructions.

The levels of Gp1bβ and Gp1bα were detected from isolated mouse platelets or bone marrow aspirates using the quantitative sandwich enzyme immunoassay technique conducted in accordance with the manufacturers protocols (ABIN424559; Antibodies-online GmbH, Aachen, Germany and SEB108Hu; USCN Life Science Inc., Wuhan, China, respectively). The optical densities at 450 nm were measured using a Biochrom ASYS UVM340 Microplate Reader (Holliston, MA).

### Statistics

Statistical significance was determined by analysis of variance (ANOVA) for multiple comparisons and the Student’s *t-*test for pair-wise comparisons. Proteomics data were also subjected to a multivariate general linear model to reveal intra-group differences. All statistical analyses were done in IBM SPSS Statistics for Mac, (ver 19.0, IBM Corp.: Armonk, NY). Statistical significance was set to *P* < 0.05. Sample sizes are given for each figure.

## Results

### Mice lacking Epac1 have prolonged *in vivo* bleeding time and *ex vivo* whole blood clotting time

**Figure 1:**
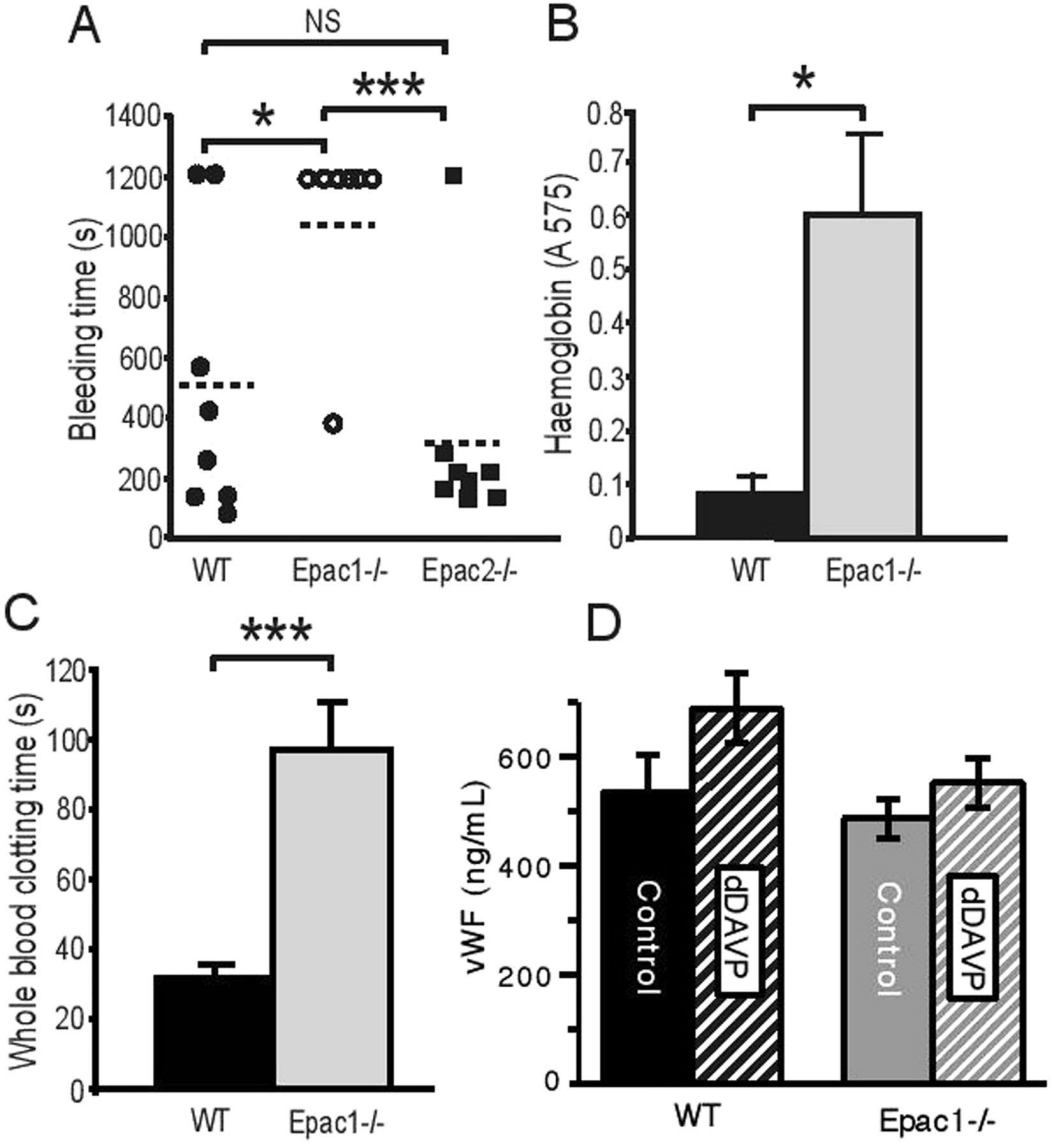
Epac1 ^−/−^ mice have increased bleeding time. (**A**) Tail bleeding time in WT (n = 8), Epac1^−/−^ (n = 7) and Epac2^−/−^ (n = 8) mice. Each point represents the measurement from one mouse. The horizontal dotted lines represent average tail bleeding time. 1200 seconds marks the experimental end point. (**B**) Blood loss quantified as amount of hemoglobin (absorbance at 575 nm) released during the tail bleeding test in WT (n = 8) and Epac1^−/−^ mice (n = 7). (**C**) Whole blood clotting time in WT (n = 6) and Epac1^−/−^ (n = 7) mice. (**D**) The plasma levels of vWF, determined by ELISA, 1.5 h after i.p. injection of 0.9% NaCl in WT (n = 4) and Epac1^−/−^ mice (n = 5), or of dDAVP (1ng/g bodyweight) in WT (n = 4) and Epac1^−/−^ mice (n = 5). The values shown are mean + /− SEM. NS = not significant, *P < 0.05, ***P < 0.005 ANOVA (A) and Student’s t-test (B, C, D).

To determine whether Epac1 or Epac2 may be required for normal hemostasis *in vivo*, we conducted a tail-bleeding test. While the median bleeding time was less than 6 minutes for both WT and Epac2^−/−^ mice, 6 out of 7 Epac1^−/−^ mice bled beyond the 20 minutes duration of the assay (Fig. 1A). Short intermittent arrest of bleeding was observed in some Epac1^−/−^ mice, but duration was less than 10 seconds. The total blood loss, assessed by its hemoglobin content, corresponded closely with the observed bleeding time, both for Epac1^−/−^ and WT mice (Fig. 1B).

The prolonged *in vivo* bleeding time could be due to altered response of the vascular endothelium and surrounding stromal tissue to the tail cutting trauma or to inherent pre-existing differences of the circulating blood, or both. To know if the Epac1^−/−^ bleeding phenotype could be reproduced *ex vivo*, we compared the clotting time of whole blood from WT and Epac1-deficient mice. The clotting time was about 3-fold prolonged in Epac1^−/−^ mice compared to WT mice, suggesting that Epac1 deficiency decreases the inherent blood clotting capacity (Fig. 1C). Epac1 appears to be involved in cAMP-regulated Weibel-Palade body exocytosis of vWF into the blood or subendothelium^6, 30^. As vasopressin is able to rapidly induce such exocytosis, we determined plasma vWF concentrations for both resting state mice and mice injected with the vasopressin analog dDVAP^6, 30^. Using ELISA, we found a non-significant trend towards lower basal vWF plasma levels in Epac1^−/−^ compared to WT mice, both for resting state animals and animals receiving dDVAP (Fig. 1D)^6, 30^. Thus, Epac1 deficiency affects whole blood clotting *ex vivo*, mainly through other mechanisms than via vWF.

### Epac1^−/−^ mice have fewer, younger and larger blood platelets that react with strong P-selectin externalization to thrombin, ADP and collagen

**Figure 2:**
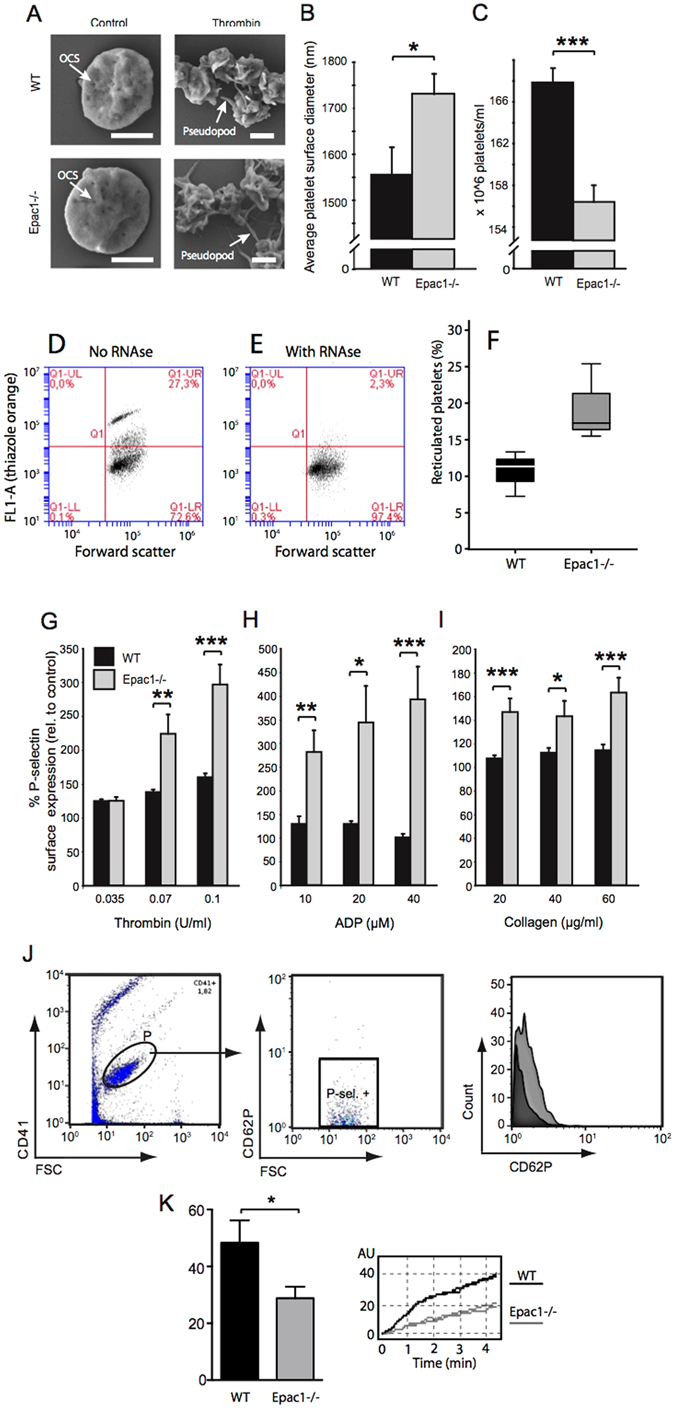
Epac1^−/−^ mice have fewer, but more reticulated platelets and more agonist-responsive blood platelets than WT mice. (**A**) Scanning electron micrographs of resting and thrombin-activated platelets from WT and Epac1^−/−^ mice. The platelets had been incubated for 10 min with vehicle (control) or 0.07 U/ml thrombin. The bars represent 1 μm. OCS: open canalicular system. (**B**) The average platelet diameter of resting platelets from WT and Epac1^−/−^ mice (WT: n = 3 mice with 28 platelet diameters measured, Epac1^−/−^ : n = 3 mice with 26 platelet diameters measured.) (**C**) Platelets from WT (n = 9) and Epac1^−/−^ (n = 10) mice were identified and counted by flow cytometry using the platelet specific marker CD41 and forward- and side scatter. (**D,E,F**) Reticulated platelet count in platelet-rich plasma from WT and Epac1^−/−^ mice. Platelets were stained with thiazole orange without (D) or with (E) RNAse treatment prior to staining. The platelets were then analyzed by flow cytometry, and the percent of reticulated platelets determined as described in the methods section. (F): Box-plot showing the reticulated platelet fraction from WT- and Epac1^−/−^ mice. The data are average of three mice from each group. P = 0.068, Student’s t-test. Platelets from Epac1^−/−^ or WT mice were exposed to various concentrations of thrombin (**G**), ADP (**H**) or collagen (**I**), and analyzed for P-selectin externalization by flow cytometry. Data shown are average + /− SEM from three independent experiments. (**J**) The gating strategy for the flow cytometric analyses, including histogram (right panel) showing a shift in mean fluorescence intensity after treatment with 0.1 U/ml Thrombin (light grey). Dark grey: unsitmulated platelets (**K**) Whole blood from WT (n = 8) and Epac^−/−^ (n = 8) mice were used in an ADP-induced aggregation assay. Data shown are average + /− SEM. OCS; open canalicular system. P; platelets. P-sel. + ; P-selectin positive. *P < 0.05, **P < 0.01, ***P < 0.005, Student’s t-test.

The platelets from Epac1^−/−^ mice had normal shape and surface morphology, as revealed by scanning electron microscopy. They had discoid shape and visible pores to the open canalicular system (OCS) in the resting state. Upon stimulation with thrombin, which caused transformation from individual platelets to aggregates, the platelets underwent the typical surface convolution with pseudopod extensions (Fig. 2A).

However, a mild macrothrombocytopenia was observed. In the resting state, Epac1^−/−^ platelets had 11% longer diameter than WT platelets (Fig. 2B), and the number of circulating platelets was slightly reduced (about 7%) in the Epac1^−/−^ mouse blood compared to WT (Fig. 2C). We also observed a higher ratio of reticulated platelets in Epac1^−/−^ mice (Fig. 2D–F), suggesting increased thrombopoiesis. However, these subtle differences are presumably of insufficient magnitude to explain the observed bleeding and clotting abnormalities in the Epac1^−/−^ mice^31, 32^.

To address this question, we investigated therefore if the Epac1^−/−^ mouse platelets had impaired sensitivity to thrombin, ADP or collagen, using agonist-induced P-selectin translocation as marker. Surprisingly, platelets derived from Epac1^−/−^ mice had increased responsiveness to all concentrations of thrombin, ADP and collagen tested (Fig. 2G–I), apart from the lowest thrombin concentration, which induced equal P-selectin translocation in platelets from Epac1^−/−^ and WT mice (Fig. 2G). The relative sensitivity of Epac1^−/−^ mouse platelets was strongest (up to 3-fold) at high ADP concentrations (Fig. 2H). In spite of this, we found a decrease in ADP-induced platelet aggregation in whole blood for the Epac1^−/−^ platelets compared to WT platelets (Fig. 2K). In conclusion, Epac1-deficient mice have a decreased number of slightly enlarged platelets with increased agonist-induced α-granule secretion, but decreased aggregation, compared to WT platelets.

### Adult and fetal Epac1^−/−^ mice have increased number of megakaryocytes

**Figure 3:**
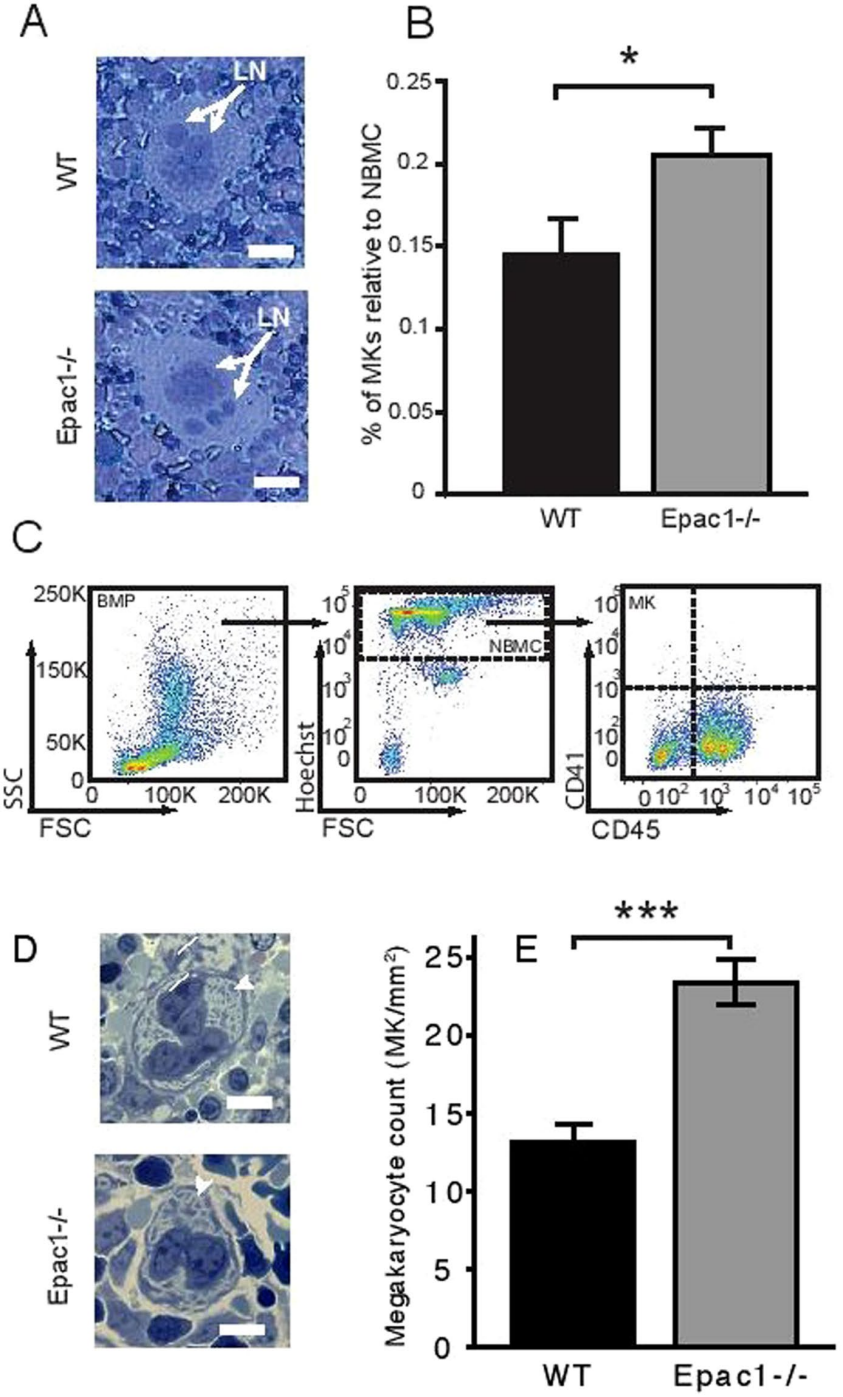
Increased number of megakaryocytes in adult bone marrow and embryonic liver. Representative micrographs of May-Grünwald Giemsa stained bone marrow megakaryocytes from WT and Epac1^−/−^ mice. (**B**) Flow cytometric determination of bone marrow megakaryocytes numbers in Epac1^−/−^ (n = 9) and WT (n = 10) mice. (**C**) Flow cytometric scatter plots with the gating strategy used to obtain data in B are shown. (**D**) Toluidine-stained sections of mouse embryonic livers showing megakaryocytes Arrowheads indicate demarcation membrane. (**E**) Number of megakaryocytes per mm^2^ in livers from WT (n = 5) and Epac1^−/−^ (n = 7) embryos (E14). Data are average and + /− SEM. LN; lobulated nucleus, BMP; bone marrow population, NBMP; nucleated bone marrow population, MK; megakaryocytes. *P < 0.05, ***P < 0.005, Student’s t-test. The bars indicate 20 μm in A and D.

We considered that the Epac1^−/−^ platelet abnormalities could be caused by altered megakaryopoiesis. The MKs from Epac1^−/−^ mice bone marrow aspirates appeared normal, with large lobulated nuclei (Fig. 3A). However, the relative MK number compared to normal bone marrow cells was higher in Epac1^−/−^ mice than in the WT mice (Fig. 3B and C). Since the liver is the main hematopoietic tissue in the embryo, we also examined the morphology and MK count in embryos harvested at 14 days after gestation. As with the bone marrow MKs, we found no apparent difference in the morphology of embryonic MKs between the Epac1^−/−^ and WT-mice. Both genotypes had typical MK morphology, which were significantly larger than the liver parenchymal cells, with lobulated nuclei, and a marked demarcation membrane^23^ surrounding a pale cytoplasm scattered with pre-platelets (Fig. 3D). In both bone-marrow smears and embryonic liver sections we failed to identify morphological features associated with malfunction, such as hyperplasia and hyperchromatic nuclei, increased polyploidy, or hypolobulated nuclei, as reviewed in^33^. Also Epac1^−/−^ embryos had higher MK count in the hematopoietic tissue compared to the WT embryo (Fig. 3E). We conclude that Epac1-deficient mice have increased number of morphologically grossly normal MKs both in fetal and adult life.

### Epac1-deficient mice have moderately altered plasma levels of several coagulation factors

**Figure 4:**
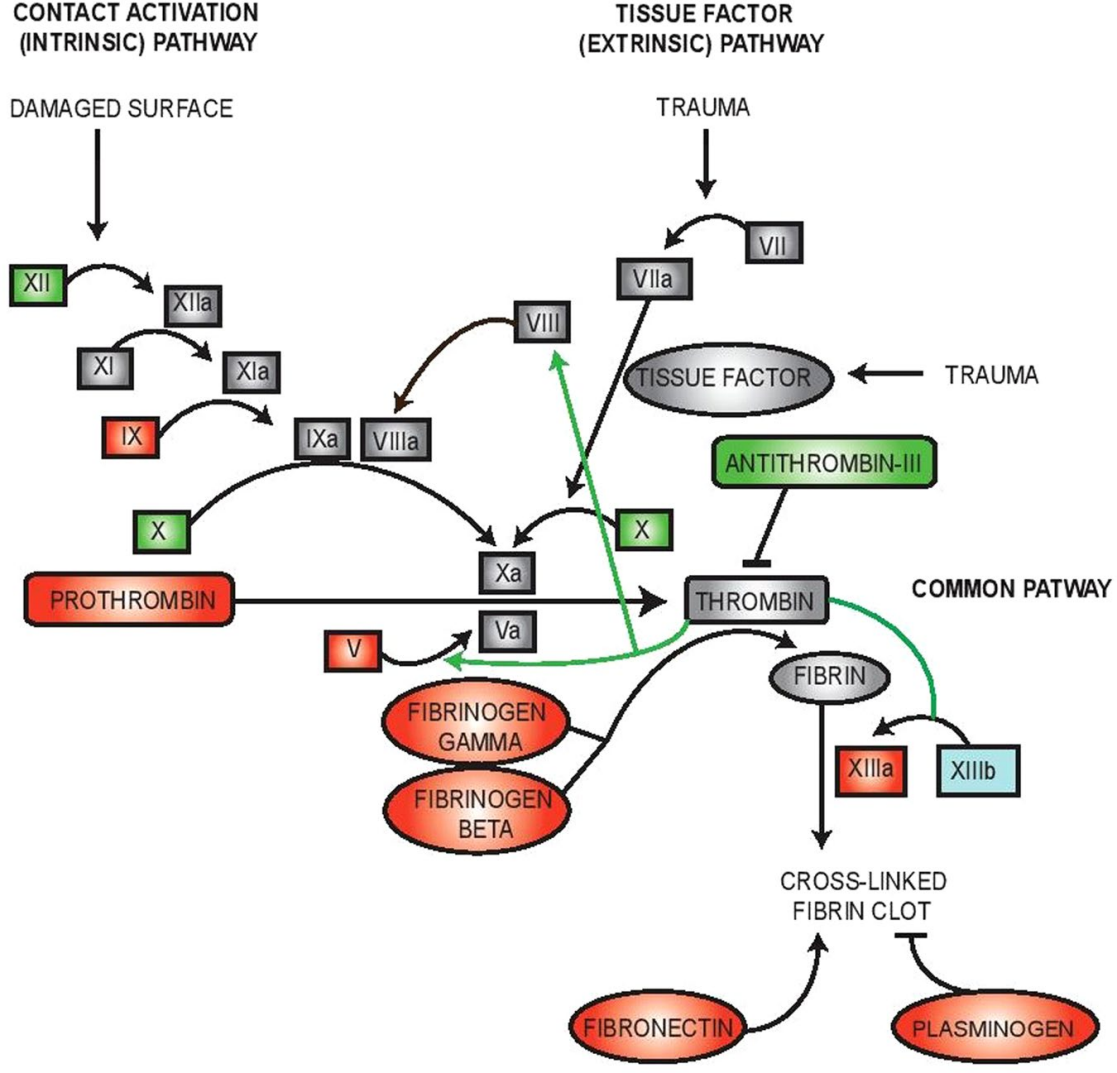
Key components of the intrinsic coagulation cascade are down-regulated in Epac1^−/−^ plasma. Plasma samples from WT (n = 6) and Epac1^−/−^ (n = 7) mice were analyzed by label-free quantitative proteomics. The differentially expressed proteins in the plasma samples were involved in the coagulation cascade and formation of fibrin clots. Down-regulated proteins are red, up-regulated are blue. Green, no altered regulation. Grey, not quantified. Thrombin signaling in this cascade is marked with green arrows.

As a consequence of platelet aggregation at sites of vascular injury, the coagulation cascade is activated, generating thrombin which stabilizes the hemostatic plug by cross-linking fibrin^34^. We analyzed therefore plasma by quantitative proteomics (see Supplementary Tables S1–S3 for all identified and quantified plasma proteins,). We detected a modest, but significant, decrease of several coagulation factors in Epac1^−/−^ plasma, including factors belonging to the intrinsic (factor IX) as well as the common (prothrombin, factor V, fibrinogen γ and β, factor XIIIa) coagulation pathways (Table 1 and Fig. 4). As the liver is the main producer of the coagulation factors, we also measured the plasma concentration of albumin. We found identical levels of albumin in Epac1^−/−^ and WT mice, suggesting that the decreased coagulation factor levels in Epac1^−/−^ mice are not a result of liver failure.

**Table 1:** Differentially regulated plasma proteins detected by proteomics.

Protein	Protein coverage (%)	Number of peptides	Ratio KO/WT	SEM	P (ANOVA)
Coagulation factor XII	28.3	29	0.96	0.0183	0.064
Coagulation factor IX	15.9	6	0.78	0.0521	0.003
Coagulation factor X	16.6	10	0.95	0.0428	0.403
Prothrombin	40.6	42	0.80	0.0169	0.000
Coagulation factor V	15.9	29	0.83	0.0236	0.000
AntiThrombin-III	51.8	55	0.94	0.0217	0.029
Fibrinogen γ	68.3	63	0.74	0.0189	0.000
Fibrinogen β	58.8	81	0.72	0.0167	0.000
Coagulation factor XIIIa	26.9	25	0.87	0.0260	0.001
Coagulation factor XIIIb	20.5	16	1.12	0.0433	0.023
Fibronectin	30.4	117	0.81	0.0119	0.000

### Epac-deficient mice have delayed rate of clot formation and decreased clot stability

**Figure 5:**
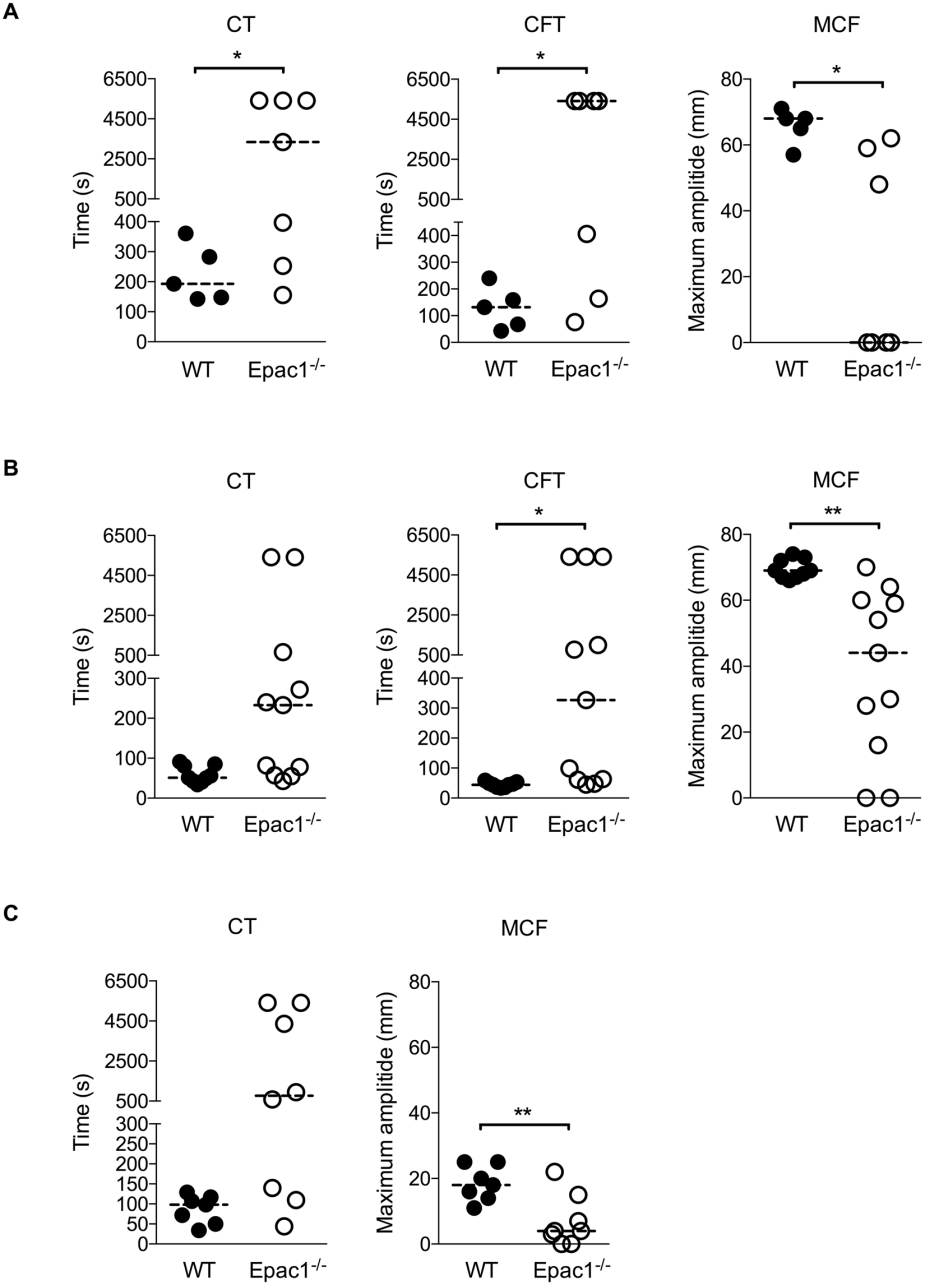
Epac1^−/−^ mice exhibit a delayed rate of clot formation and fragile clot stability *ex vivo*. ROTEM characterization of the coagulation process in citrated whole blood from WT and Epac1^−/−^ mice. Activated CT: clotting time i.e., the latency until the clot reaches a firmness of 2 mm, CFT: clot formation time and MCF: maximum clot firmness in mm was assessed by ROTEM assays (**A**) INTEM, (**B**) EXTEM and (**C**) FIBTEM. In FIBTEM assays the actin polymerization and platelet activation blocker cytochalasin D is present. Each point represents the measurements of one mouse. The horizontal dotted lines represent the median, n = 5–11 mice per group. *P < 0.05, **P < 0.01, Student’s t-test.

During the tail-bleeding test on Epac1^−/−^ mice we noticed that bleeding ceased and then rapidly re-commenced several times, indicating the formation of short-lived, abortive hemostatic plugs. We conducted rotational thromboelastometry (ROTEM) assays to assess the clot strength and stability in whole blood, where platelets and coagulation factors are present together in physiological relevant proportion. Samples from WT and Epac1^−/−^ mice were compared by INTEM (Fig. 5A), EXTEM (Fig. 5B) and FIBTEM (Fig. 5C) analysis to test the function of the primary and the secondary hemostasis. We found that the clotting time (CT), defined as the time from start of activation to start of coagulation, was prolonged for Epac1^−/−^ samples for both tests. The same trend was observed for the rate of blood clot formation (CFT, clot formation time until 20 mm amplitude reached). The maximum clot firmness (MCF) was lower for Epac1^−/−^ compared to WT samples. When a poor EXTEM is compared to a normal FIBTEM, this would indicate deficient platelet function or low platelet numbers. To compare CT and MCF without the influence of platelets we assessed FIBTEM in the presence of the actin polymerization and platelet activation blocker cytochalasin D. Under such conditions, even with only one platelet inhibitor^35^ the FIBTEM data for Epac1^−/−^ mice showed very low values for MCF, thus suggesting that without platelets present there will be very little or no clot formation. The FIBTEM assay is highly affected by the expression level of fibrinogen and factor XIII, which we found significantly down-regulated in the proteomics data (Table 1 and Fig. 5). Thus, our results suggests that the defects in blood clotting for Epac1^−/−^ mice are caused by impaired secondary hemostasis.

### Platelets from Epac1^−/−^ deficient mice have increased ILK and imbalanced expression of important GPIb-IX-V receptor proteins

To search for an explanation for the apparently contradictory α-granule activation reactivity of Epac1^−/−^ platelets (Fig. 2D–F) and the prolonged bleeding (Fig. 1A) we used label-free quantitative proteomics on platelet samples. We found that several proteins involved in platelet activation were differentially expressed in Epac1^−/−^ and WT mice derived platelets (Table 2 and Supplementary Table S4). We found that Epac1^−/−^ platelets had up-regulated αIIbβ3-associated protein Integrin linked protein kinase (ILK). This kinase is required for agonist-induced α-granule secretion, which was increased in the platelets from our Epac1^−/−^ mice (Fig. 2), and is known to be dramatically decreased in ILK-deficient mice^36^.

**Table 2:** The impact of Epac1 deletion on expression of ILK and GPIb-related platelet adhesion proteins, detected by proteomics.

Protein	Part of/associated with complex	Unique peptides	Intensity WT	Intensity Epac1^−/−^	Ratio KO/WT	PEP value
GPIbα	GPIb-IX-V	5	9373800	9188200	0.98	2.21E-10^6^
GPIbβ	GPIb-IX-V	2	7048600	919540	0.13	3.68E-72
GPIX	GPIb-IX-V	1	1253500	n.f.	—	0.0013581
GPV	GPIb-IX-V	6	5321700	16134000	3.03	1.57E-79
ILK	Integrin αIIbβ3	5	1686400	6406100	3.80	2.73E-12

ILK, integrin linked protein kinase, GP, glycoprotein.

Another finding was strong down-regulation of the transmembrane protein GPIbβ, with unaltered expression of the GPIbα (Table 2) and 3-fold increase of GPV (Table 2). In platelets, 2 GP1bβ subunits are linked through disulfide bonds to 1 GPIbα subunit, forming a complex known as GPIb, which in turn interacts noncovalently with GPIX and GPV to generate the glycoprotein (GP) Ib-IX-V receptor complex^37^. The expression of GPIbα subunit was not altered, whilst the expression of GPV subunit was 3-fold increased. The GPIX subunit was only identified in the WT fraction, and could thus not be quantified.

**Figure 6:**
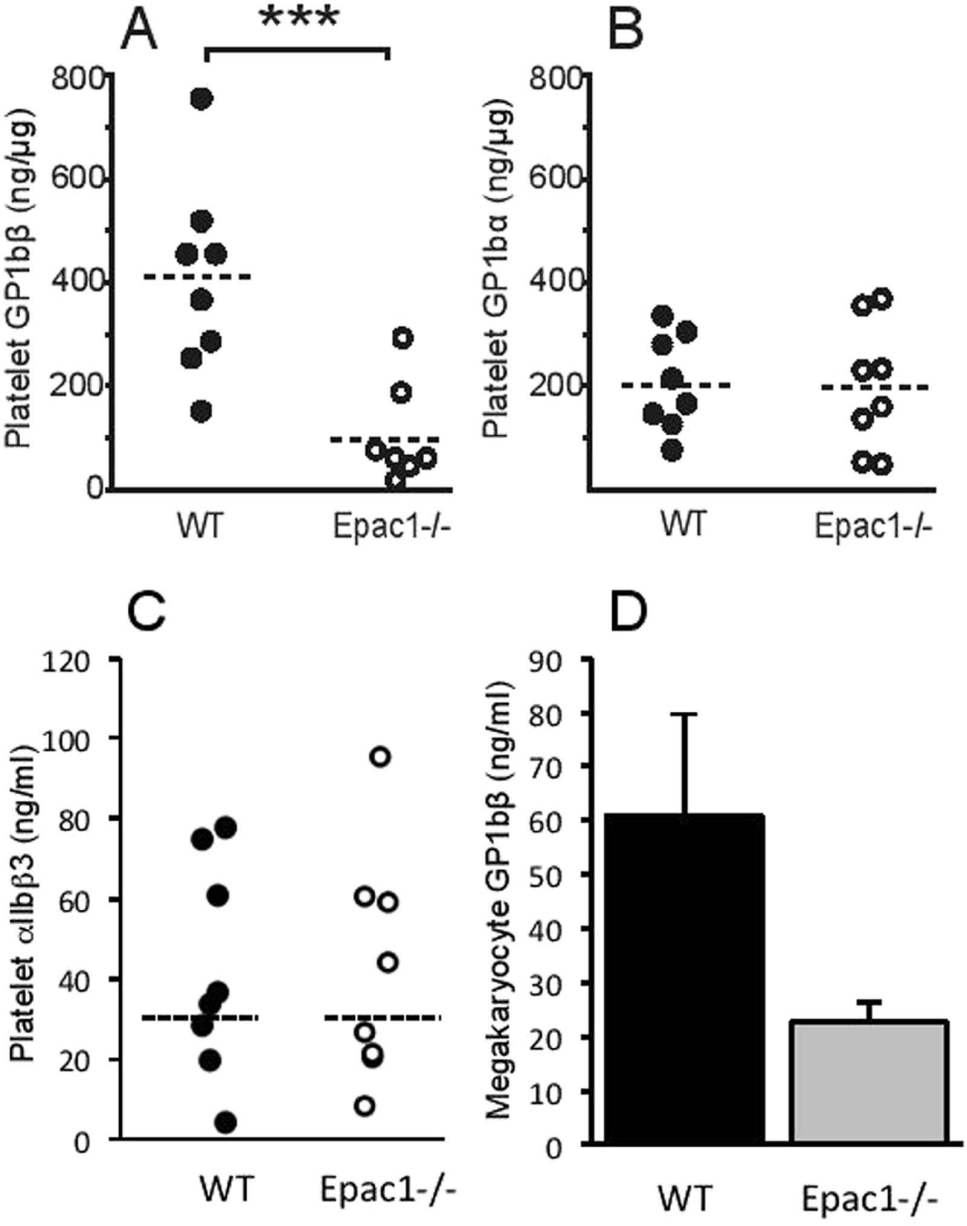
Decreased platelet levels of GP1bβ and un-altered levels of GP1bα in Epac1−/− mice. Quantification of GP1bβ (**A**), GP1bα (**B**) and αIIbβ3 (**C**) of platelets from WT (n = 8) and Epac1^−/−^ (n = 8) mice by ELISA. Each dot represents the data from a single mouse. (**D**) Quantification of megakaryocyte GP1bβ by ELISA from WT (n = 3) and Epac1^−/−^ (n = 3) mice. The horizontal dashed lines represent the mean. ***P < 0.005.

Quantitative sandwich ELISA assays helped verify the proteomic data and establish the stoichiometry of GP1bβ and GP1bα. In accordance with the proteomic data, the ELISA assay targeting GP1bβ showed a 4-fold down-regulation in Epac1^−/−^ platelets compared to WT (Fig. 6A), while the ELISA assay targeting GP1bα showed similar levels of GP1bα in WT and Epac1^−/−^ platelets (Fig. 6B). The ELISA assays also enabled quantification of the GP1bβ and GP1bα concentrations, and we found the WT platelet concentration of GP1bβ to be double that of the WT and Epac1^−/−^ platelet concentrations of GP1bα. The WT, but not the Epac1^−/−^, platelet concentrations of GP1bβ and GP1bα is in accordance with the known formation of the GPIb complex, where the ratio of these subunits is 2:1, respectively^37^.

We estimated also the megakaryocyte GPIbβ levels by ELISA of bone marrow extract. The GPIbβ content was lower in Epac1^−/−^ derived bone marrow extract (Fig. 6D) suggesting that the deficiency was present already during megakaryopoiesis, and that Epac1 is required already at this stage to support full GPIbβ expression. Since platelet aggregation depends on αIIbβ3, we quantified also the expression of αIIbβ3 by ELISA. As shown in Fig. 6C, similar levels of αIIbβ3 were observed in Epac1 and WT derived platelets.

## Discussion

The present study demonstrates that Epac1^−/−^ mice have: (1) prolonged bleeding time; (2) impaired clotting connected to secondary hemostasis; (3) fewer, larger and more *in vitro* P-selectin responsing platelets; (4) increased number of reticulated platelets (5); increased number of MKs in adult bone marrow and embryonic liver (6); decreased aggregatory platelets response in whole blood. Several of these observations can be explained by the feedback loop between the platelets and their megakaryocyte precursors^38, 39^. If the demand for circulating platelets increases, for instance following blood loss or increased consumption of platelets, the MKs proliferate to produce platelets to compensate the loss. These newly produced platelets are larger and more reactive than older circulating platelets^40, 41^.

To probe the molecular basis for our observations, we performed label-free quantitative proteomics on plasma and platelet samples to identify proteins with irregular expression in Epac1^−/−^ mice. Several proteins involved in the coagulation cascade (coagulation factor IX, V, XIIIa, prothrombin) and the cross-linking fibrin clot formation system (fibrinogen γ and β, fibronectin) were moderately downregulated in Epac1^−/−^ mice plasma samples, compatible with an intravascular coagulation (DIC)-like effect with increased consumption of coagulation factors^42^. While these findings are expected consequences of increased bleeding, it is not obvious how they occur in the first place. The Epac1^−/−^ mice breed normally, are healthy and apparently indistinguishable from WT littermates. Their fecal matter shows no hemoglobin, and there are no signs of petechiae on skin or mucous membranes to suggest spontaneous bleeding. Thus, it seems it is only after being challenged that their bleeding phenotype becomes evident.

We found differentially expressed proteins also in the Epac1^−/−^ platelet samples. They belong to, or are associated with, platelet receptors (ILK, GPIbβ, GPV). The platelet receptor protein GPIbβ, which is drastically down-regulated in the Epac1^−/−^ platelets, is part of the adhesion platelet receptor complex GPIb-IX-V. The interaction between GPIb-IX-V and ECM-associated vWF is sufficiently rapid to allow for transient binding of platelets under conditions of high shear stress^43^. Deficient or nonfunctioning GPIb-IX-V is characteristic of the inherited bleeding disorder BSS^44^, which shares several of the symptoms observed in the Epac1^−/−^ mice, such as increased bleeding time, lower platelet counts and larger platelets^4, 45, 46^. Decreased platelet counts and increased platelet size in patients with BSS have been reported to be similar regardless of which GPIb-IX-V -subunit is affected. Also, in accordance with our findings for Epac1^−/−^ mice (Figs 1A–C, 2C), the bleeding diathesis in BSS patients has been shown to be more severe than expected from the platelet counts^47^. In line with this, a study by Kato *et al*. clearly shows that genetic deletion of mouse platelet GP1bβ*-*component produces a Bernard-Soulier phenotype. The GP1bβ knockout mice exhibited a severe bleeding phenotype, macrothrombocytopenia and contained enlarged α-granules^48^. Our thromboelastometry analysis (INTEM and EXTEM) showed that 1) the rate of blood clot formation in Epac1^−/−^ mice is delayed and 2) the clot is smaller and the clot stability is more fragile (lower MCF) compared to WT mice (Fig. 5). MCF is an indirect index of blood clot retraction and thus related to platelet contractile force. Reduction in platelet contractile force has been shown to correlate with reduced expression of GP1bβ and postoperative blood loss for cardiac surgery patients^49, 50^. The difference between WT and Epac1^−/−^ mice regarding clot retraction persisted when platelet activation was blocked, suggesting that also other aspects than platelet contraction contributed to the deficiency. The low values for CT and MCF with the FIBTEM test for Epac1^−/−^ mice indicate that the plasma levels of fibrinogen and factor XIII may be responsible for the observed dysfunction of the clotting process. Furthermore, the decreased abundance of fibrinogen may contribute to the observed inhibited aggregatory effect in Epac1^−/−^ mice. Thus, the defects in blood clotting for Epac1^−/−^ mice seems to be connected to secondary hemostasis.

GPIbα is the main ligand binding subunit of GPIb-IX-V, with both GPIbβ and GPIX regulating its function and expression. Studies by Strasser *et al*. show that covalent binding between GPIbβ and GPIbα is necessary to render the vWF binding sites of GPIbα functional^44^, and others report that the intracellular region of GPIbβ rather than GPIbα is critical for vWF-induced signaling^51, 52^. Strasser *et al*. also suggest that GPIbβ is required for stabilizing GPIX, which seems highly susceptible to proteolytic degradation when GPIbβ is not present^44^. The fact that GPIX was not detected in the Epac1^−/−^ platelets is in accordance with such a mechanism. However, we found GPIbα levels in Epac1^−/−^ platelets to be unaltered. Both GPIX and GPIbβ are reported to be required for efficient plasma membrane expression of GPIbα^53^. GPIbβ and GPIX interact and together act as a chaperone for GPIbα, transporting it through the membrane systems of the maturing megakaryocyte and onto the surface of newly formed platelets^44^. The non-regulated level of GPIbα in combination with the severely down-regulated GPIbβ reported here is puzzling and indicates that Epac1 may act more broadly than by decreasing GPIbβ in the megakaryocytes.

The GPV subunit was up-regulated in the Epac1^−/−^ platelets, but this protein is not necessary for GPIbα and GPIbβ expression and function in platelets^54^. Interestingly, GPV knockout mice have shorter bleeding time and hyper-responsive platelets^55^. Thus, GPV seems to be a negative modulator of platelet function, and may contribute to the observed bleeding phenotype in our Epac1^−/−^ mice.

The binding of vWF to the GPIb-IX-V complex initiates a signal that culminates in the activation of the integrin receptor αIIbβ3^56, 57^. Interestingly, the αIIbβ3-associated protein ILK is up-regulated in Epac1^−/−^ mice. ILK induces conformational changes in the extracellular part of αIIbβ3 that results in higher affinity for fibrinogen and vWF, thus facilitating platelet-platelet interactions and aggregation^36, 58, 59^. While ILK deficient platelets have decreased agonist-induced α-granule secretion^36^, our Epac1^−/−^ platelets have increased agonist-induced α-granule secretion (Table 2), in accordance with their increased ILK level. The up-regulated ILK and α-granule secretion levels may be a compensatory response for malfunctioning GPIb-IX-V adhesion *in vivo*.

Taken together, these results indicate that there may be a direct relationship between platelet levels of ILK and α-granule secretion. The increased α-granule secretion in Epac1^−/−^ platelets stands in contrast to exocytosis studies in other cell types, where Epac1 has been found to be required for secretion^6^. A direct link between Epac1 and “inside-out” Rap1-mediated signaling through other integrin receptors than αIIbβ3 has been established in several cell types, such as monocytes^11^ and ovarian carcinoma cells^60, 61^. However, we have previously used the well-known Epac1 agonist 8-pCPT-2′-O-Me-cAMP to study RAP1 activation in human platelets^14^. In sharp contrast to our results from other cell types^62, 63^, this agonist completely failed to activate platelet RAP1^14^. This result, taken together with comprehensive proteomic and transcriptomic analysis from other groups, strongly suggests that Epac1 is not present in platelets^11, 12^. Thus, we believe that our findings with regards to platelet function and signaling in the Epac1^−/−^ mice is not a direct result of loss of Epac1 activity in platelets. Moreover, as platelets are anucleate and have limited synthesis of new proteins, the differential expression of proteins in the Epac1^−/−^ platelets probably arises from the lack of Epac1 signaling in other cells of the hemopoietic platelet lineage. Our results showing that the level of GBIbβ is also reduced in the megakaryocytes supports this. Furthermore, Epac has important functions in several cell types of the vascular niche, including CD34^+^ hematopoietic stem cells, mesenchymal stem cells and pluripotent stem cells^16, 64^. Crucial roles of Epac signaling in hematopoietic cell generation were recently demonstrated^16^. Moreover, developmental differences in megakaryopoiesis and platelet generation can be determined by the bone marrow niche, including stromal cells and gradient of cytokines and growth factors^65^. Thus, regulation of megakaryopoiesis and functional platelet synthesis is a complex process regulated at multiple phases in which we here show that Epac1 plays a key role. To our knowledge, we here provide the first direct evidence of an important role for Epac1 in secondary hemostasis. Our data also suggests a model where Epac1 is required for normal megakaryopoiesis and the subsequent expression of several proteins involved in key platelet functions, but where Epac1 itself is not present nor directly participates in signaling in mature circulating platelets.

## Electronic supplementary material

Supplementary Dataset 1
